# *In vitro* evaluation of N-acetylcysteine, calcium hydroxide, chlorhexidine digluconate and their combinations effect against five dental obligate anaerobic bacteria

**DOI:** 10.7717/peerj.21831

**Published:** 2026-10-22

**Authors:** Lara Steffany de Carvalho, Júlia Andrade de Oliveira, Gabriel Manente Cezarino, Luiz Augusto Rodrigues dos Santos, Gustavo Guimarães Guerrero, Mauro Felipe Rios do Nascimento, Marcia Carneiro Valera, Luciane Dias de Oliveira, Amjad Abu Hasna

**Affiliations:** 1 Institute of Science and Technology, Department of Restorative Dentistry, São Paulo State University, São José dos Campos, São Paulo, Brazil; 2 Institute of Science and Technology, Department of Restorative Dentistry, Endodontics Division, São Paulo State University, São José dos Campos, São Paulo, Brazil; 3 School of Dentistry, Department of Oral and Maxillofacial Surgery, Prosthodontics and Traumatology, University of São Paulo, São Paulo, São Paulo, Brazil; 4 Institute of Science and Technology, Department of Biosciences and Oral Diagnosis, São Paulo State University, São José dos Campos, São Paulo, Brazil

**Keywords:** Acetylcysteine, Calcium hydroxide, Chlorhexidine, Anaerobic bacteria

## Abstract

**Background:**

This study aimed to evaluate the antibacterial action of N-acetylcysteine (NAC), calcium hydroxide Ca(OH)_2_, chlorhexidine digluconate (CHX), and their combinations against five obligate anaerobic bacteria including *Fusobacterium nucleatum, Porphyromonas endodontalis, Porphyromonas gingivalis, Prevotella intermedia*, and *Parvimonas micra* in planktonic and biofilm forms.

**Methods:**

Eight experimental groups were evaluated in this study including saline solution (SS) (control group), Ca(OH)_2_, CHX, NAC, NAC + Ca(OH)_2_, NAC + CHX, Ca(OH)_2_ + CHX, and NAC + Ca(OH)_2_ + CHX. The anti-planktonic evaluation was performed by determination of minimum inhibitory concentration (MIC) and minimum bactericidal concentration (MBC) following Clinical and Laboratory Standards Institute guidelines (CLSI M11). The antibiofilm was evaluated by 3-(4,5-dimethylthiazol-2-yl)-2,5-diphenyltetrazolium bromide (MTT) and crystal violet (CV) assays. Data were analyzed statistically considering a significant level of (*α* ≤ 0.05).

**Results:**

It was found that the MIC and MBC values of the tested intracanal medications ranged from 1.95–500 mg/mL. MTT and CV assays showed significant, species-dependent antibiofilm activity. CHX was most effective against *F. nucleatum*; NAC-based medications, especially combined with Ca(OH)_2_ and/or CHX, were superior against *P. endodontalis, P. intermedia*, and *P. micra*. Ca(OH)_2_-containing groups were particularly effective against *P. gingivalis*. Therefore, NAC + Ca(OH)_2_ and NAC + Ca(OH)_2_ + CHX can be considered as effective combinations of intracanal medication against different obligate anaerobic bacteria.

## Introduction

Primary endodontic infections are characterized by a complex polymicrobial community, predominantly composed of obligate anaerobic bacteria (Siqueira & Rôças, 2009). These communities are largely dominated by Gram-negative species, including *Fusobacterium nucleatum*, *Porphyromonas endodontalis*, *Porphyromonas gingivalis*, and *Prevotella intermedia*, as well as Gram-positive bacteria, including *Parvimonas micra* and *Streptococcus* species (Siqueira & Rôças, 2022). The predominance of obligate anaerobes is largely attributed to the low oxygen concentrations within the root canal system. Nevertheless, the microbial composition of primary endodontic infections may vary considerably among studies and between individuals, as bacterial phylotypes and their distribution are influenced by multiple factors, including geographic location, sex, and ethnicity (Louzada et al., 2020; Machado et al., 2020).

Conventionally, root canal treatment (RCT) relies on the removal of necrotic and infected tissues through instrumentation, based on the principles of cleaning and shaping of the root canal system, as proposed by Schilder (1974), using endodontic files synergistically with irrigating solutions (Khoury et al., 2024b). Despite technological advances, including the development of engine-driven endodontic file systems (Ribeiro Camargo et al., 2020; Dos Reis et al., 2023; Ragozzini et al., 2024; Castillo Marin et al., 2024; Da Silva et al., 2025), the efficacy of the RCT remains limited by the complex anatomy of the root canal system, including ramifications and isthmuses that hinder complete contact between files, irrigants, and canal walls, allowing microbial persistence, regrowth, and retention of bacterial byproducts (Lui et al., 2023). In parallel, increasing microbial resistance further compromises effective disinfection, highlighting the need for intracanal medications (ICM) to enhance canal disinfection and detoxification (Carvalho et al., 2020; Oliveira et al., 2022; De Oliveira et al., 2024).

Calcium hydroxide (Ca(OH)_2_) is the most widely used ICM because of its antimicrobial activity associated with high pH and hydroxyl ion release, as well as its ability to neutralize bacterial endotoxins (Mohammadi, Shalavi & Yazdizadeh, 2012). However, its efficacy may be limited by low diffusibility and the dentin buffering effect (Zand et al., 2017). Chlorhexidine digluconate (CHX) 2% gel is also widely used because of its broad-spectrum antimicrobial activity, prolonged substantivity, and efficacy in the presence of blood and exudate (Mohammadi, 2008). Nevertheless, despite its efficacy against planktonic bacteria and superficial biofilms, CHX shows limited penetration into deeper areas of dentinal tubules (Arathi et al., 2019).

N-acetylcysteine (NAC), an aminothiol compound derived from L-cysteine, has gained increasing attention in Endodontics due to its antimicrobial activity and its ability to disrupt biofilm formation, maturation, and stability (Quah et al., 2012; Abu Hasna et al., 2020; Abu Hasna et al., 2025). *In vitro* studies have demonstrated its efficacy against several endodontic pathogens, including *Enterococcus faecalis* and *Escherichia coli* and endotoxin of *E. coli* (Martinho et al., 2023; Khoury et al., 2024a) supporting its potential as a sole ICM, or in combination with other agents.

As these ICM have different mechanisms of antimicrobial action, combining two or more agents may improve their antimicrobial potential against obligate anaerobic pathogens. In addition, to the best of our knowledge, NAC as ICM has not yet been tested against obligate anaerobic bacteria of endodontic interest. Therefore, the aim of this study was to evaluate the antibacterial and antibiofilm action of Ca(OH)_2_, CHX, NAC, and their combinations against five obligate anaerobic bacteria *F. nucleatum*, *P. endodontalis*, *P. gingivalis*, *P. intermedia,* and *P. micra* in both planktonic and biofilms forms. The null hypothesis is that these ICM would have no antibacterial or antibiofilm effect against the tested pathogens.

## Materials & Methods

### Experimental groups

A total of eight experimental groups were evaluated in this study:

• Saline solution (SS): (negative control group) sterile saline solution (Eurofarma, São Paulo, SP, Brazil) was used to confirm bacterial growth.
• Ca(OH)_2_: a paste was obtained by mixing 100 mg of Ca(OH)_2_ powder (Biodinâmica Química e Farmacêutica LTDA, Paraná, Brazil) with 100 µL of sterile saline solution.
• CHX: 2% chlorhexidine digluconate gel (Terapêutica Farmácia de Manipulação, São José dos Campos, SP, Brazil) used at a volume of 100 µL.
• NAC: a paste was obtained by mixing 100 mg of NAC powder (Merck KGaA, Darmstadt, Germany) with 100 µL of sterile saline solution.
• NAC + Ca(OH)_2_: a paste was prepared by mixing 50 mg of NAC and 50 mg of Ca(OH)_2_ powders with 100 µL of sterile saline solution.
• NAC + CHX: a paste was prepared by mixing 100 mg of NAC powder with 100 µL of 2% CHX gel.
• Ca(OH)_2_ + CHX: a paste was prepared by mixing 100 mg of Ca(OH)_2_ powder with 100 µL of 2% CHX gel.
• NAC + Ca(OH)_2_ + CHX: a paste was prepared by mixing 50 mg of NAC and 50 mg of Ca(OH)_2_ powders with 100 µL of 2% CHX gel.

All formulations were freshly prepared immediately before each experiment using standardized powder-to-vehicle proportions and identical mixing procedures to obtain a homogeneous dentifrice-like consistency.

### Anti-planktonic evaluation by determination of minimum inhibitory concentration and minimum bactericidal concentration

Independent, monotype suspensions of five bacterial strains including *F. nucleatum* (ATCC 25586), *P. endodontalis* (ATCC 35406), *P. gingivalis* (ATCC 33277), *P. intermedia* (ATCC 33563), and *P. micra* (ATCC 23195) were prepared and standardized for the experiments.

The bacterial strains were reactivated in enriched Brucella broth (BD, Heidelberg, Germany) supplemented with 1% hemin and 1% vitamin K (menadione) (Sigma-Aldrich, St. Louis, MO, USA) and incubated anaerobically for 48 h in a Whitley DG250 anaerobic workstation (Don Whitley Scientific Limited, West Yorkshire, UK). Anaerobic conditions were continuously monitored using anaerobic indicator strips to verify maintenance of the oxygen-free environment throughout the experiments.

Subsequently, the cultures were streaked onto Brucella agar plates (Himedia, Mumbai, India) supplemented with sheep blood, 1% hemin and 1% vitamin K (menadione), then incubated anaerobically for an additional 48 h.

Bacterial colonies were then suspended in sterile saline solution, and the turbidity of each suspension was adjusted spectrophotometrically at 600 nm. The suspensions were then standardized to a final concentration of 1 × 10^6^ CFU/mL according to the Clinical and Laboratory Standards Institute guidelines for anaerobic bacteria (CLSI M11).

For the broth microdilution assay, 100 µL of each freshly prepared medication was added to the first well of each row of a 96-well microplate, while the control wells received 100 µL of sterile saline solution, using a conventional calibrated air-displacement micropipette. Subsequently, 100 µL of Brucella broth was added to each first well, and the mixture was thoroughly homogenized by repeated pipetting before preparing 10 consecutive twofold serial dilutions. A total of 100 µL of the standardized bacterial suspension (1 × 10^6^ CFU/mL) was then added to each well, and the plates were incubated anaerobically at 37 °C for 24 h. Each minimum inhibitory concentration (MIC) and minimum bactericidal concentration (MBC) assay was performed in triplicate using the same standardized bacterial inoculum.

The MIC was defined as the lowest concentration of ICM that showed no visible turbidity, indicating inhibition of bacterial growth, was observed. To confirm the MIC results, aliquots (10 µL) were collected from wells showing no visible turbidity and from the well corresponding to the MIC value. These aliquots were streaked onto Brucella agar plates (Himedia, Mumbai, India) supplemented with sheep blood 1% hemin and 1% vitamin K (menadione), then incubated under anaerobic conditions at 37 °C for 48 h. Later, the plates were examined for bacterial growth. The MBC was defined as the lowest concentration of each ICM at which no colony growth was observed, indicating complete bacterial killing.

### Evaluation of antibiofilm action using metabolic activity and biomass assays

The antibiofilm activity of the ICMs was evaluated against mono-species biofilms of the same bacterial strains used in the planktonic assays. Five independent bacterial suspensions were prepared and standardized spectrophotometrically to approximately 1 × 10^8^ CFU/mL in sterile saline solution (0.9% NaCl) (Eurofarma, São Paulo, SP, Brazil). The standardized suspensions were used exclusively for initial bacterial adhesion, whereas biofilm growth and maturation occurred during the subsequent incubation period. Subsequently, 100 µL of each bacterial suspension was added to the wells of 96-well microplates and incubated at 37 °C under agitation (75 rpm) for 90 min to allow initial bacterial adhesion.

After the adhesion period, the supernatant was discarded, 100 µL of Brucella broth was added to each well, and the plates were incubated anaerobically at 37 °C for 7 days for biofilm maturation, with renewal of the culture medium every 48 h. Following biofilm formation, the biofilms were exposed to the experimental ICMs for 24 h under anaerobic conditions at 37 °C. Biofilm maturation was performed according to a previously validated protocol involving seven days of anaerobic incubation with culture medium renewal every 48 h, which has consistently been shown to produce mature mono-species biofilms (Abu Hasna et al., 2022b; Abu Hasna et al., 2022a).

Biofilm metabolic activity was subsequently assessed using the 3-(4,5-dimethylthiazol-2-yl)-2,5-diphenyltetrazolium bromide (MTT) assay. Two independent experiments were performed, each containing six replicates per experimental group, resulting in a total sample size of *n* = 12.

Following the contact period with the experimental materials, 100 µL of MTT solution [3-(4,5-dimethylthiazol-2-yl)-2,5-diphenyltetrazolium bromide] (Sigma-Aldrich, St. Louis, MO, USA) was added to each well. The plates were incubated protected from light under anaerobic conditions at 37 °C for 1 h. After incubation, the MTT solution was removed, and 100 µL of dimethyl sulfoxide (DMSO) (Sigma-Aldrich, St. Louis, MO, USA) was added to solubilize the formazan crystals. The plates were incubated at 37 °C for 10 min and placed on a constant shaker for an additional 10 min.

Absorbance was measured at 570 nm using a microplate reader (BioTek Instruments Inc., Winooski, VT, USA). The optical density (OD) values were converted into percentages of viable cells according to the following formula:

\begin{eqnarray*}\text{Biofilm metabolic activity}= \frac{\text{Mean OD of control group}-\text{OD of treated group}}{\text{Mean of OD of control group}} \times 100. \end{eqnarray*}

Furthermore, biofilm biomass was quantified using the crystal violet (CV) assay. The microplates of each bacterial inoculum were interpreted separately. Following the 24 h contact period with the experimental ICMs, biofilms were fixed by adding 200 µL of methanol per well for 20 min. The methanol was removed, and the plates were incubated at 37 °C for 24 h. Subsequently, 200 µL of 1% (v/v) crystal violet (CV) solution was added to each well for 5 min. Excess dye was removed, and the wells were gently washed with sterile saline solution. The retained dye, corresponding to the total biofilm biomass (including bacterial cells and extracellular biofilm matrix) was solubilized by adding 33% (v/v) acetic acid (Abu Hasna et al., 2022b; Abu Hasna et al., 2022a). Optical density was measured using a microplate reader, and the results were expressed as percentages of biofilm biomass reduction using the formula:

\begin{eqnarray*}\text{Biofilm biomass}= \frac{\text{Mean OD of control group}-\text{OD of treated group}}{\text{Mean of OD of control group}} \times 100. \end{eqnarray*}

### Statistical analysis

The MIC and MBC values were determined from the consensus of the triplicate measurements and are presented descriptively; therefore, no inferential statistical analysis was performed for these assays. However, data of MTT and CV were tabulated and assessed by Shapiro–Wilk normality test. Then, data were analyzed by Kruskal-Wallis test and followed by Dunn’s *post hoc* test because data did not follow a normal distribution. A significant level of 5% (*p* < 0.05) was adopted using GraphPad Prism 10.6.1 (GraphPad Software, San Diego, CA, USA).

## Results

### MIC and MBC results

In this study, no detectable MIC and MBC values were observed for the control group of saline solution, indicating the absence of both bacteriostatic and bactericidal activity. In contrast, CHX and NAC demonstrated antibacterial activity at relatively low concentrations, ranging from 1.95–7.81 mg/mL and 1.95–31.25 mg/mL, respectively. These findings highlight their efficacy against the tested obligate anaerobic pathogens when compared with Ca(OH)_2_, which required substantially higher concentrations (62.5–500 mg/mL) to achieve inhibitory and bactericidal effects.

The combined effects of NAC + Ca(OH)_2_ and NAC + CHX were generally limited, as effective inhibition or killing of planktonic obligate anaerobic bacteria occurred only at concentrations of 62.5–500 mg/mL and 31.25–62.5 mg/mL, respectively. An exception was observed against *P. endodontalis*, in which the NAC + CHX combination exhibited enhanced activity at lower concentrations (1.95–3.9 mg/mL).

Conversely, the combinations of Ca(OH)_2_ + CHX and NAC + Ca(OH)_2_ + CHX showed superior antibacterial performance, with efficacy observed from 3.9 mg/mL onward. Moreover, for the remaining tested bacteria, all evaluated concentrations of these combinations were sufficient to inhibit or eradicate the obligate anaerobic pathogens as shown in Table 1.

**Table 1:** MIC and MBC values of the tested intracanal medications against the tested obligate anaerobic pathogens.

	*F. nucleatum*		*P. endodontalis*		*P. gingivalis*		*P. intermedia*		*P. micra*	
	MIC	MBC	MIC	MBC	MIC	MBC	MIC	MBC	MIC	MBC
Saline solution	–	–	–	–	–	–	–	–	–	–
Ca(OH)_2_	62.5	62.5	250	500	250	500	250	500	250	500
CHX	3.9	3.9	1.95	3.9	3.9	3.9	1.95	3.9	3.9	7.81
NAC	15.62	15.62	15.62	31.25	1.95	3.9	7.81	15.62	3.9	7.81
NAC + Ca(OH)_2_	62.5	62.5	250	500	62.5	62.5	250	500	62.5	62.5
NAC + CHX	62.5	62.5	1.95	3.9	62.5	62.5	31.25	31.25	31.25	31.25
Ca(OH)_2_+ CHX	3.9	3.9	3.9	3.9	All	3.9	3.9	3.9	All	3.9
NAC + Ca(OH)_2_+ CHX	All	3.9	All	3.9	All	3.9	3.9	3.9	All	3.9

### Evaluation of antibiofilm action using biofilm metabolic activity—MTT assay

Against *F. nucleatum*, all tested ICM significantly reduced biofilm metabolic activity compared with the control group (*P* < 0.0001), with CHX showing the greatest antibiofilm effect and differing significantly from the other groups (*P* < 0.0040). Against *P. endodontalis*, only NAC and the combination NAC + Ca(OH)_2_ + CHX were effective in reducing biofilm metabolic activity (*P* = 0.0071 and *P* = 0.0155, respectively). For *P. gingivalis*, groups containing Ca(OH)_2_ showed significantly greater reductions in biofilm metabolic activity compared with the control group (*P* < 0.0001), whereas the remaining treatments did not differ from the control. Against *P. intermedia*, NAC, NAC + CHX, and NAC + Ca(OH)_2_ + CHX significantly reduced biofilm metabolic activity compared with both the control and the other tested groups (*P* = 0.0316, *P* = 0.0215 and *P* = 0.0388, respectively). For *P. micra*, the combination NAC + Ca(OH)_2_ promoted the greatest reduction in biofilm metabolic activity, differing significantly from all other groups (*P* < 0.0001); nevertheless, all remaining treatments also reduced biofilm metabolic activity when compared with the control group (*P* < 0.0001) as shown in Fig. 1.

**Figure 1:**
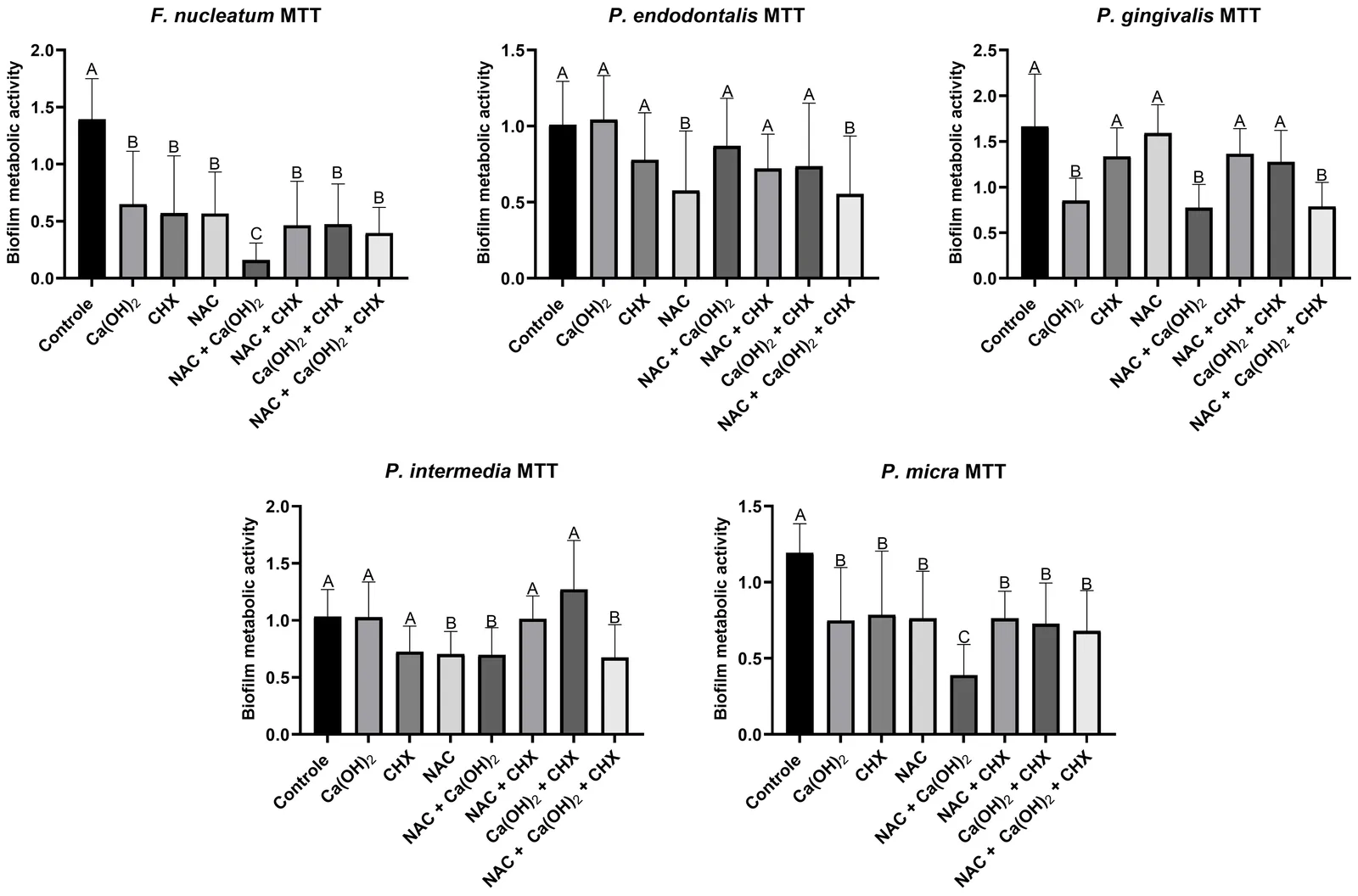
Biofilm metabolic activity of the tested bacterial species after treatment with different intracanal medications, as determined by the MTT assay. Different uppercase letters (A, B, and C) indicate statistically significant differences among the experimental groups (*P* < 0.05).

### Evaluation of antibiofilm action using biofilm biomass—CV assay

For *F. nucleatum*, only NAC, CHX, and the NAC + CHX combination significantly reduced biofilm biomass compared with the other groups (*P* < 0.0001). Against *P. endodontalis*, NAC, NAC + CHX, and NAC + Ca(OH)_2_ + CHX were effective in reducing biofilm biomass, with statistically significant differences compared with the remaining treatments (*P* < 0.0001, *P* = 0.003, and *P* = 0.0121, respectively). In *P. gingivalis*, all experimental groups significantly reduced biofilm biomass compared with the control group (*P* < 0.0001), with no significant differences among treatments. Against *P. intermedia*, all tested groups significantly reduced biofilm biomass compared with the control group (*P* < 0.0001), except for Ca(OH)_2_ and Ca(OH)_2_ + CHX. Finally, for *P. micra*, NAC + Ca(OH)_2_ + CHX showed the most pronounced reduction in biofilm biomass, differing significantly from the other treatments (*P* = 0.0002) as shown in Fig. 2.

**Figure 2:**
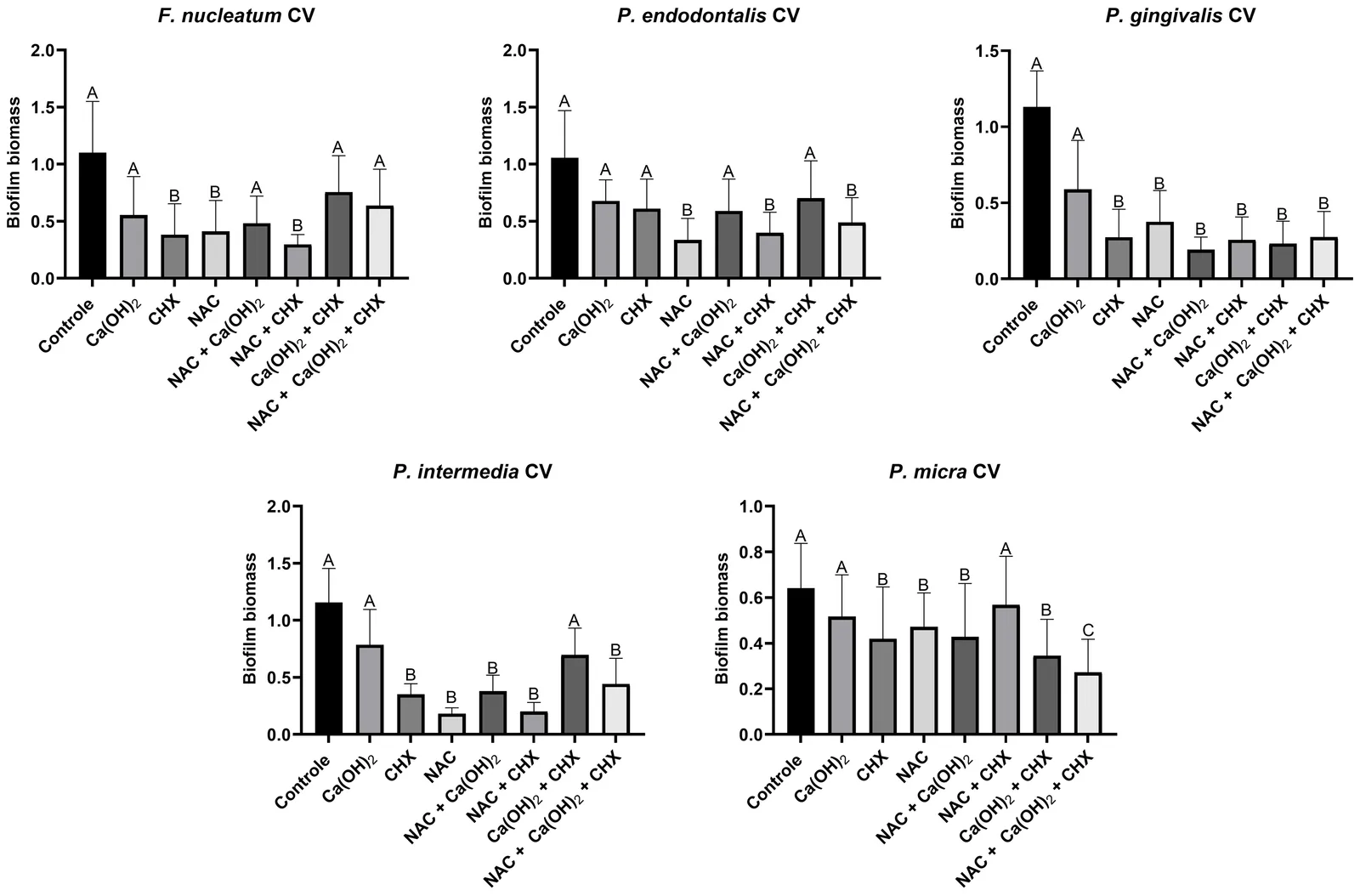
Biofilm biomass of the tested bacterial species after treatment with different intracanal medications (crystal violet assay). Different uppercase letters (A, B, and C) indicate statistically significant differences among the experimental groups (*P* < 0.05).

## Discussion

The use of ICM during the RCT is often indicated in different situations, mainly because of their antimicrobial properties, especially in infected cases. In this study, it was found that NAC, Ca(OH)_2_ and CHX and their combinations demonstrated antibacterial and/or antibiofilm activity against at least one of the tested obligate anaerobic pathogens. Therefore, the null hypothesis of this study that the tested ICM would have no antibacterial or antibiofilm effect against the tested pathogens was partially rejected, as the antibacterial efficacy varied according to the ICM and bacterial species tested.

The rationale behind testing the effect of the ICM against these obligate anaerobic pathogens is that Gram-negative species, such as *F. nucleatum*, *P. endodontalis*, *P. gingivalis*, and *P. intermedia*, as well as Gram-positive bacteria, including *P. micra* and *Streptococcus* species, are commonly found in the root canal system of teeth with primary endodontic infection (Siqueira & Rôças, 2009; Siqueira & Rôças, 2022). Furthermore, these pathogens are used when the focus is testing ICMs, endodontic irrigants or bioceramic cements against obligate anaerobic pathogens of endodontic interest (Abu Hasna et al., 2022b; Abu Hasna et al., 2022a; De Sá Assis et al., 2022). The experimental formulations were designed based on pilot data obtained before performing the experiments of this study, as well considering previous studies evaluating NAC, Ca(OH)_2_, and CHX as ICM (Quah et al., 2012; Abu Hasna et al., 2020; Calefi et al., 2022; Khoury et al., 2024a). The proportions used in the combination groups were selected to provide equal contributions of the powder components while maintaining a standardized powder-to-vehicle ratio and a homogeneous paste consistency suitable for laboratory testing.

To the best of our knowledge, based on the available literature, no previous study has evaluated the antibacterial effect of NAC, Ca(OH)_2_ and CHX and their combinations against obligate anaerobic bacteria of endodontic interest. In this study, the saline solution group was not bacteriostatic or bactericidal against the planktonic form of the tested bacteria, conforming its suitability as a negative control and demonstrating that bacterial growth occurred in the absence of antimicrobial agents, as reported in previous studies (Abu Hasna et al., 2022b; Abu Hasna et al., 2022a).

In the study of Quah et al. (2012), it was found that the MIC and MBC of NAC were 1.56 and 12.5 mg/mL, respectively, against *E. faecalis,* and in another study, the values were 2.5 and 20 mg/mL, respectively against the same bacteria (Ulusoy et al., 2016). The values in the present study were proximal as MIC and MBC ranged between 1.95–31.25 mg/mL against the tested obligate anaerobic bacteria. One point to be observed in the present study is that the addition of Ca(OH)_2_ or CHX did not make it more effective in lower concentrations, as MIC and MBC values of NAC + Ca(OH)_2_ and NAC + CHX groups were 62.5–500 mg/mL and 31.25–62.5 mg/mL, respectively. Nevertheless, the addition of both Ca(OH)_2_ and CHX to NAC resulted in superior antibacterial efficacy observed from 3.9 mg/mL onward.

A study limitation should be highlighted here, in which the checkerboard assays were not performed; and subsequently, the optimal concentrations of the combined formulations and their potential synergistic, additive, or antagonistic interactions were not investigated. Therefore, future studies should consider using checkerboard methodology to identify the lowest effective concentrations of these combinations and to further characterize their antimicrobial interactions.

Against biofilms, NAC demonstrated antibacterial efficacy at even lower concentrations against the tested obligate anaerobic bacteria, as evidenced by biofilm metabolic activity (MTT assay) and biomass (CV assay), further supporting its potent and concentration-independent antibiofilm activity. Similar findings were reported by Abu Hasna et al. (2020), who showed that NAC significantly reduced CFU/mL counts and biofilm load using confocal laser scanning microscopy (CLSM) at a concentration of one mg/mL (Abu Hasna et al., 2020). In addition, in another study NAC alone was more effective than Ca(OH)_2_ against *E. faecalis* biofilms, as demonstrated by live/dead staining under CLSM at a concentration of 500 mg/0.5 mL of propylene glycol with four serial dilutions (Calefi et al., 2022). Nonetheless, a more recent investigation found no statistically significant differences between NAC and Ca(OH)_2_ regarding their effects against *E. faecalis* and *Escherichia coli*, as well as on *E. coli* LPS levels after 7 and 14 days, with both agents showing comparable antibacterial and antiendotoxin activity as assessed by CFU/mL counting (Khoury et al., 2024a).

In the present study, the combination of NAC with Ca(OH)_2_ enhanced antibacterial activity against all tested obligate anaerobic bacteria in both MTT and CV assays, except for *P. endodontalis*, by greater reductions in biofilm metabolic activity (MTT assay) and biofilm biomass (crystal violet assay). Consistent with these findings, previous study (Khoury et al., 2024a) reported that the association of NAC and Ca(OH)_2_ resulted in reduced CFU/mL counts of *E. faecalis* and *E. coli*, as well as decreased levels of *E. coli* lipopolysaccharide levels after 7 days of treatment. Conversely, other investigations have shown that the addition of Ca(OH)_2_ to NAC does not further enhance its antibacterial efficacy against *E. faecalis* biofilms following a 7-day exposure period, indicating that the efficacy of this combination may be species- and biofilm-dependent (Adl et al., 2022). Although the present findings indicate that the NAC + Ca(OH)_2_ combination has promising antibacterial and antibiofilm activity *in vitro*, the clinical relevance of these reductions remains to be established. In the clinical setting, reducing biofilm metabolic activity and biomass may contribute to lowering the microbial burden within the root canal system, potentially improving disinfection before obturation.

When comparing NAC with CHX, previous studies have shown that NAC, at concentrations ranging from 12.5–100 mg/mL, was more effective than a 2% CHX solution against multispecies biofilms composed of *Actinomyces naeslundii*, *Lactobacillus salivarius*, *Streptococcus mutans*, and *E. faecalis* after 24 h of exposure (Moon et al., 2016). Similar findings were reported in a scanning electron microscopy study, in which NAC at concentrations of 25–100 mg/mL and a 7-day exposure period was more effective than CHX in eradicating multispecies endodontic biofilms (Choi et al., 2018). Furthermore, NAC at a concentration of 200 mg/mL demonstrated superior antibiofilm activity compared with 2% CHX when both were used as irrigants (five mL for 5 min), as assessed by CLSM against *E. faecalis* and *S. mutans* biofilms (Bhasin et al., 2019). In the present study, NAC showed antibacterial activity comparable to CHX against *F. nucleatum*, *P. gingivalis*, and *P. micra* in both MTT and CV assays, by reducing the biofilm metabolic activity and biomass, respectively, while exhibiting superior antibacterial efficacy against *P. endodontalis* in the same assays.

The combination of Ca(OH)_2_ and 2% CHX gel was reported to be more effective than NAC alone when used as an ICM after a 14-day period in a clinical study evaluating root canals from patients diagnosed with primary endodontic infection and apical periodontitis, in which approximately 40 bacterial species were identified (Martinho et al., 2023). In contrast, in the present *in vitro* study, NAC alone demonstrated equal or superior antibacterial activity against the tested obligate anaerobic bacteria after 24 h, as assessed by MTT and CV assays, by reducing the biofilm metabolic activity and biomass, respectively. These discrepancies may be attributed to fundamental differences between clinical and *in vitro* experimental conditions, including microbial diversity, biofilm maturity, host-related factors, and treatment duration.

In the MTT assay, which reflects the metabolic activity of viable biofilm cells, the association of NAC with Ca(OH)_2_ demonstrated enhanced antibacterial effects against *F. nucleatum* and *P. micra*. Although the present study did not investigate the underlying mechanisms, previous studies have suggested that NAC may interfere with biofilm stability by disrupting the extracellular polymeric substance (EPS) and reduce biofilm cohesion (Da Silva et al., 2024), whereas Ca(OH)_2_ exerts its antibacterial activity through hydroxyl ion release and a highly alkaline pH (Estrela et al., 2023). Therefore, the improved antibacterial activity observed with this combination may be related to the complementary mechanisms of these agents. However, this hypothesis should be confirmed in future mechanistic studies.

In the CV assay, which evaluates total biofilm biomass, the triple combination of NAC + Ca(OH)_2_ + CHX showed the greatest reduction in biofilm biomass against *P. micra*. Although the mechanisms responsible for this finding were not investigated in the present study, previous reports have shown that NAC may reduce biofilm stability (Da Silva et al., 2024), Ca(OH)_2_ exhibits antibacterial activity through hydroxyl ion release (Mohammadi & Dummer, 2011), and CHX acts on cell membrane permeability and substantivity (Mahendra et al., 2014), leading to more pronounced biofilm detachment and biomass reduction. Therefore, the superior performance of the triple combination may reflect the complementary actions of these agents rather than a single mechanism. Further studies are required to elucidate the biological mechanisms underlying these observations.

Another point to be highlighted, is that the MTT and CV assays are complementary one to another regarding the antibiofilm activity of the tested ICM. The MTT assay reflects the metabolic activity of viable cells through the reduction of tetrazolium salts to formazan, whereas the CV assay quantifies the total biofilm biomass, including viable and non-viable bacterial cells as well as the extracellular polymeric matrix. Consequently, reductions in metabolic activity do not necessarily correspond to proportional reductions in biofilm biomass. This was truly confirmed in the present study while interpreting the obtained resulta. For, *P. gingivalis*, all experimental groups, except for Ca(OH)_2_ reduced biofilm biomass, while producing only limited reductions in metabolic activity, suggesting that the formulations may have primarily disrupted biofilm structure without substantially affecting the biofilm viability. Conversely, *F. nucleatum* exhibited reduced biofilm metabolic activity across all treatments, whereas reductions in biofilm biomass were observed only for selected ICM, indicating that bacterial metabolism may have been impaired despite partial preservation of the biofilm matrix.

Lastly, the combinations of NAC + Ca(OH)_2_ and NAC + Ca(OH)_2_ + CHX demonstrated enhanced antibacterial and antibiofilm activity against *F. nucleatum* and *P. micra*, as evidenced by reductions in biofilm metabolic activity and biomass. These pathogens have significant clinical relevance due to their role in biofilm formation and the establishment of primary periapical infections (Horiuchi et al., 2020). These findings suggest that such combined ICM may represent a promising strategy for targeting these pathogens and other obligate anaerobic bacteria. Nevertheless, clinical studies are strongly warranted to validate these *in vitro* study using mono-species biofilm models, which do not fully reproduce the complexity and inter-individual variability of polymicrobial biofilms associated with primary endodontic infections. Therefore, future investigations should include multispecies biofilm models, particularly mixed biofilms involving *F. nucleatum* and *P. micra*, to better simulate clinical conditions.

## Conclusions

NAC + Ca(OH)_2_ and NAC + Ca(OH)_2_ + CHX were effective in reducing the biofilm metabolic activity and biomass of *F. nucleatum* and *P. micra* under the experimental conditions of this *in vitro* study. These findings support the potential of NAC-containing formulations as ICM against obligate anaerobic endodontic pathogens under the experimental conditions evaluated. Further studies using multispecies biofilm models and clinical investigations are required to confirm their potential as ICM.

##  Supplemental Information

10.7717/peerj.21831/supp-1 Supplemental Information 1 Raw data of MTT assay

10.7717/peerj.21831/supp-2 Supplemental Information 2 Raw data of violet crystal assay

10.7717/peerj.21831/supp-3 Supplemental Information 3 Raw data of MIC and MMC

## Additional Information and Declarations

### Data Availability

The following information was supplied regarding data availability: The data is available in the Supplemental Files. The data is available at: Abu Hasna A. 2026. Estudo *in vitro* da ação antimicrobiana, da atividade antitoxina, da citotoxicidade, da genotoxicidade e da atividade anti-inflamatória da N-acetilcisteína, do hidróxido de cálcio e da clorexidina, isolados e em associação [Data set]. Universidade Estadual Paulista (Unesp). https://hdl.handle.net/11449/318572.
