# Supplementary material for: In vitro evaluation of N-acetylcysteine, calcium hydroxide, chlorhexidine digluconate and their combinations effect against five dental obligate anaerobic bacteria
Source: PeerJ. 2026 Oct 22;14:e21831. doi: 10.7717/peerj.21831 (PMC13695961; doi:10.7717/peerj.21831)
Supplement: Supplementary file 1 [file peerj-14-21831-s003.pdf]

1 Determination of minimum inhibitory concentration (MIC) and minimum microbicidal concentration (MMC)

Fusobacterium nucleatum	MIC	MMC
Saline solution	-	-
$Ca(OH)_2$	62.5	62.5
CHX	3.9	3.9
NAC	15.62	15.62
NAC + $Ca(OH)_2$	62.5	62.5
NAC + CHX	62.5	62.5
$Ca(OH)_2$ + CHX	All concentration were effective	3.9
NAC+ $Ca(OH)_2$ + CHX	All concentration were effective	3.9

Porphyromonas gingivalis	MIC	MMC
Saline solution	-	-
$Ca(OH)_2$	500	250
CHX	3.9	3.9
NAC	1.95	3.90
NAC + $Ca(OH)_2$	62.5	62.5
NAC + CHX	62.5	62.5
$Ca(OH)_2$ + CHX	All concentration were effective	3.9
NAC+ $Ca(OH)_2$ + CHX	All concentration were effective	3.9

Parvimonas micra	MIC	MMC
Saline solution	-	-
$Ca(OH)_2$	500	250
CHX	3.9	19.5
NAC	3.9	19.5
NAC + $Ca(OH)_2$	62.5	62.5
NAC + CHX	31.25	31.25
$Ca(OH)_2$ + CHX	All concentration were effective	3.9

NAC+ $Ca(OH)_2$ + CHX	All concentration were effective	3.9

Porphyromonas endodontalis	MIC	MMC
Saline solution	-	-
$Ca(OH)_2$	500	250
CHX	1.95	3.9
NAC	31.25	15.62
NAC + $Ca(OH)_2$	500	250
NAC + CHX	1.95	3.9
$Ca(OH)_2$ + CHX	3.9	3.9
NAC+ $Ca(OH)_2$ + CHX	All concentration were effective	3.9

Prevotella intermedia	MIC	MMC
Saline solution	-	-
$Ca(OH)_2$	500	250
CHX	1.95	3.9
NAC	15.62	7.81
NAC + $Ca(OH)_2$	500	250
NAC + CHX	31.25	15.62
$Ca(OH)_2$ + CHX	3.9	3.9
NAC+ $Ca(OH)_2$ + CHX	3.9	3.9

Legend: $Ca(OH)_2$: calcium hydroxide. CHX: chlorhexidine. NAC: N-acetylcysteine.
